# Cytomegalovirus infection in HIV-infected versus non-infected infants and HIV disease progression in Cytomegalovirus infected versus non infected infants early treated with cART in the ANRS 12140—Pediacam study in Cameroon

**DOI:** 10.1186/s12879-017-2308-x

**Published:** 2017-03-23

**Authors:** Anfumbom K. W. Kfutwah, Paul Alain T. Ngoupo, Casimir Ledoux Sofeu, Francis Ateba Ndongo, Georgette Guemkam, Suzie Tetang Ndiang, Félicité Owona, Ida Calixte Penda, Patrice Tchendjou, Christine Rouzioux, Josiane Warszawski, Albert Faye, Mathurin Cyrille Tejiokem

**Affiliations:** 1 Virology Service, Centre Pasteur of Cameroon, Member of the International Network of Pasteur Institutes, P.O. Box 31076, Yaounde, Cameroon; 2 Epidemiology and Public Health Service, Centre Pasteur of Cameroon, Member of the International Network of Pasteur Institutes, Yaounde, Cameroon; 3 Pediatric Day Clinic, Mother and Child Center of the Chantal Biya Foundation, Yaounde, Cameroon; 4 Pediatric Service, Hospital Center Essos, Yaounde, Cameroon; 5 Day Clinic, Laquintinie Hospital, Douala, Cameroon; 6 Faculty of Medicine and Pharmaceutical Sciences, University of Douala, Douala, Cameroon; 7 Assistance Publique des Hôpitaux de Paris, Laboratoire de Virologie, Hôpital Necker, Paris, France; 8 Université Paris 5 René Descartes, URF de Médecine, Paris, France; 9 Equipe 4 (VIH et IST)—INSERM U1018 (CESP), Le Kremlin Bicêtre, France; 10 Assistance Publique des Hôpitaux de Paris, Service d’Epidémiologie et de Santé Publique, Hôpital de Bicêtre, Le Kremlin Bicêtre, France; 11 Université de Paris Sud 11, Paris, France; 12 Assistance Publique des Hôpitaux de Paris, Pédiatrie Générale, Hôpital Robert Debré, Paris, France; 13 Université Paris 7 Denis Diderot, Paris Sorbonne Cité, Paris, France; 14 INSERM UMR 1123, ECEVE, Paris, France; 15 P.O. Box 1274, Yaounde, Cameroon

**Keywords:** Cytomegalovirus, Human Immunodeficiency Virus, Co-infection, Early treated HIV-infected infants

## Abstract

**Background:**

The outcome of CMV/HIV co-infection in infants treated early with combined antiretroviral therapy (cART) in resource-limited settings has not been described.

We aimed to estimate the prevalence and identify factors associated with early CMV infection in HIV-infected and non-infected infants included in a study in Cameroon, and to compare HIV disease progression and survival after 1 year of early cART, following infants’ CMV status.

**Methods:**

HIV-infected infants followed from birth or from HIV diagnosis before 7 months old and HIV-uninfected infants born to HIV-infected or uninfected mothers were tested for CMV at a median age of 4.0 months [Interquartile range (IQR): 3.4–4.9]. Multivariable logistic regression was performed to identify factors associated with CMV infection. Early cART was offered to HIV-infected infants: mortality, immunological and virological outcomes were assessed.

**Results:**

Three hundred and sixty-nine infants were tested. The proportion of infants infected with CMV at baseline was significantly higher in HIV-infected than in HIV-uninfected groups (58.9% (86/146) vs 30.0% (67/223), *p* < 0.001). At baseline, median CMV viral load was higher in HIV-infected (3.7 log copies/ml [IQR; 3.1–4.3]) than in HIV-uninfected infants (2.8 log copies [IQR; 2.1–3.4], *p* < 0.001). cART was initiated in 90% of HIV-infected infants (132/146) at a median age of 4.0 months (IQR; 3.2–5.9); in this sub-group CMV infection was independently associated with being followed from the time of HIV diagnosis rather than from birth (aOR = 3.1, 95%CI [1.2–8.0]), born to a non-single mother (aOR = 3.4[1.4–8.1]), and breastfeeding (aOR = 7.3 [2.7–19.4]). HIV-infected infants were retested after a median of 7.1 months [4.8–9.5]: CMV was undetectable in 37 of the 61 (60.7%) initially CMV-infected cases and became detectable in 8 of the 38 (21.1%) initially CMV-negative cases. After 1 year of cART, the probability of death (0.185 vs 0.203; *p* = 0.75), the proportion of cases with HIV RNA viral load <400 copies/ml (75.5% vs 61.5%; *p* = 0.17) and the mean CD4 percentage increase (10.97% vs 6.88%; *p* = 0.15) did not differ between CMV+ and CMV- infants.

**Conclusions:**

We observed a high prevalence of CMV infection among HIV-infected infants. Early initiation of cART may have limited the negative impact of CMV even in the absence of specific anti-CMV treatment.

## Background

African children infected with human cytomegalovirus (CMV) usually acquire it during the first year of life [1]. It is one of the major congenital infections worldwide and a significant opportunistic pathogen for immune-compromised individuals [2, 3]. In most immunocompetent infants, CMV infection is asymptomatic; however, HIV-infected children are at risk of developing CMV disease [4]. Several studies report that HIV-infected newborns are at higher risk of symptomatic congenital CMV infection than uninfected newborns. CMV may act as a cofactor for HIV disease progression, and the risk of infant mortality is increased in HIV/CMV co-infected infants [5–7].

The consequences of prenatal antiretroviral (ARV) prophylaxis on CMV transmission from HIV-infected women to their babies and of highly active antiretroviral therapy (HAART) to halt the progression of CMV disease are still unclear. In the French perinatal cohort, the incidence of vertical CMV transmission from HIV-infected mothers declined with the introduction of HAART [7]. Nevertheless, no significant decrease was observed in the prevalence of congenital CMV infection in children born to HIV-infected mothers receiving prenatal antiretroviral therapy in a recent study, [8]. Little is known about congenital CMV infection among HIV-exposed infants in sub-Saharan Africa [9–11].

We estimated the prevalence of early CMV infection in HIV-infected and HIV-uninfected infants included before 7 months of age in three urban hospitals in Cameroon. We identified risk factors and compared disease progression and survival during the first year after early cART following CMV status.

## Methods

### The ANRS 12140—Pediacam study

The ANRS 12140-Pediacam is an ongoing prospective observational cohort coordinated at the Centre Pasteur of Cameroon, designed to assess the feasibility of early cART in HIV-infected infants in three referral hospitals: the Mother and Child Center of the Chantal Biya Foundation (MCC-CBF), the Hospital Center Essos (HCE) both in Yaounde; and Laquintinie Hospital (LH) in Douala. Infant inclusion into the Pediacam was conducted in two consecutive phases. The first phase, planned from birth to 14 weeks, included all infants born live to HIV-infected mothers and an equivalent number of infants born to HIV-uninfected mothers matched individually on gender and recruitment site. The newborn pairs were followed, tested for HIV (where needed) and given routine vaccinations.

All identified HIV-infected infants (Group 1i) and selected controls of uninfected infants followed from birth, born to HIV-infected mothers (Group 1ni) or to HIV-uninfected mothers (Group 2ni) were included in the second phase that was planned for children from 14 weeks to 2 years old. Inclusion into the second phase was also offered to HIV-infected children not included from birth but aged less than 7 months at diagnosis of HIV infection (Group 3i). After inclusion in the second phase, infants underwent biological assessment including CMV testing and HIV-infected infants were offered systematic cART according to the Cameroonian guidelines at the time of this study. First-line treatment was zidovudine (or stavudine for infants with anemia) and lamivudine associated with lopinavir/ritonavir if nevirapine (NVP) had been used for the Prevention of mother-to-child transmission of HIV (PMTCT), or with NVP if it had not been used for PMTCT. Biological analyses (including whole blood counts, CD4 T-lymphocyte cell counts, liver function tests, amylases, blood sugar levels, tuberculosis and malaria) were subsequently planned every 3 months or every 6 months until 2 years of age for HIV-infected and HIV-uninfected infants, respectively.

### Study population

All the infants participating in the ANRS 12140-Pediacam prospective study and screened for CMV infection at inclusion into the second phase were considered in the present analysis.

**Fig. 1:**
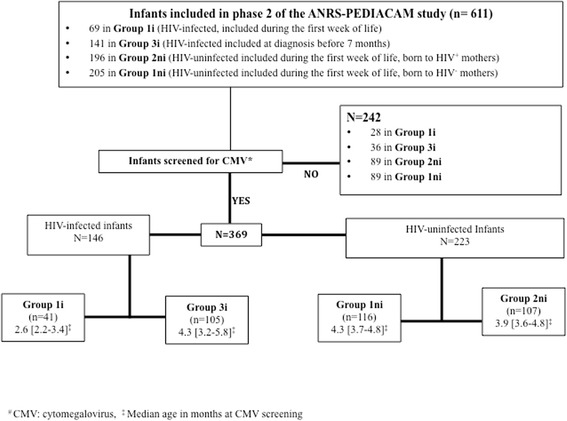
Study population and description of infant age at CMV screening in the phase 2 of the ANRS-PEDIACAM study, Cameroon, 2007–2012

From November 2007 to October 2013, a total of 611 infants were included in the second phase of the ANRS 12140-Pediacam study: 69 were HIV-infected followed from birth (Group 1i); 141 were HIV-infected not followed from birth and diagnosed before age 7 months (Group 3i); 205 were HIV-uninfected born to HIV-infected mothers (Group 1ni); and 196 were HIV-uninfected born to HIV-uninfected mothers (Group 2ni) (Fig. 1). Of these infants, 242 were not screened for CMV, including 140 (57.9%) in Douala where there was no system in place to collect and transfer samples to Centre Pasteur of Cameroon in Yaounde for analysis, and 102 in Yaounde.

### Outcomes definition and covariates

The main outcome was the CMV infection status defined as the presence or absence of any CMV genomes in the blood at inclusion into the second phase of the ANRS 12140-Pediacam study. The CMV test was repeated at various time points during the first year of antiretroviral treatment to assess disease progression.

Secondary outcomes were measured during the year following initiation of antiretroviral therapy: Kaplan-Meier probability of death, viral suppression defined as viral load <400 copies/mL at two consecutives routine tri-monthly visits, and mean difference in CD4 percentage difference between inclusion and the last follow-up measure.

Risk factors of CMV infection recorded included those that were related to infant characteristics both at birth and at inclusion, maternal characteristics and socio-economic status, quality of antenatal care and obstetrical context at delivery.

### Cytomegalovirus diagnosis and quantification

CMV testing was planned at inclusion into the second phase and every 3 or 6 months according to infant HIV status (see above). At each time point, 200 μL of whole blood was drawn and stored at -80 °C for CMV analysis. Viral nucleic acids (DNA) were extracted using the QIAamp DNA mini kit (QIAGEN®, Hilden, Germany) and eluted from the columns with 200 μL of DNAse/RNAse free water. Aliquots of 5 μL were used for PCR. Primers and probe for the real-time PCR quantification corresponded to the UL 123 exon 4 gene as previously described [12, 13]. A plasmid containing the amplified sequence of strain AD 169 (Tebu France) was used as an external standard. For each batch, serial dilutions of plasmid (range, 50 to 500,000 copies) were amplified to establish a standard curve and allow the quantification of CMV in clinical samples. Appropriate negative and positive controls were included in each run. The results are reported as the number of CMV genome copies/mL of whole blood.

### Statistical analysis

Maternal socio-economic and demographic data, antenatal care and obstetrical context, and infant characteristics are described using proportions for categorical variables, or medians and interquartile ranges for continuous variables. Bivariable analysis was used to study associations between baseline factors and CMV infection, both overall and according to infant HIV status. Multivariable logistic regression models were used to identify independent factors, including initially non-collinear covariates associated with CMV infection (as a dependent variable) if the *p*-value was ≤ 0.25 in the bivariable analysis. The final model was obtained by successively removing variables not associated at a *p*-value <0.05 and only if the odds ratios for the remaining variables were unchanged and taking interactions into account. Known risk factors (breastfeeding) and sampling variables (infant group) were maintained in the final model.

Among HIV-infected children treated with cART, the relation between CMV status and various outcomes of HIV disease progression, censored at 52 +/-6 weeks after ART initiation, was studied. The risk of death or death/loss to follow up in the two CMV status groups was estimated by Kaplan-Meier survival analysis using log rank test to test for significance. The proportion of infants with adequate viral load suppression and mean difference of CD4 percentage were compared between the two groups using Fisher’s exact tests and Student’s t test, respectively. All statistical analyses were performed using R 2.15 software.

### Ethical considerations

The ANRS 12140-PEDIACAM study received ethical approval in Cameroon from the National Ethics Committee. Administrative authorization was obtained from the Ministry of Public Health before the start of the study. Written informed consent from one of the parents or guardians was required for inclusion into either phase 1 or phase 2 of the ANRS-Pediacam study.

## Results

### Characteristics of the study population by infant group

Of the 611 infants included in the second phase of Pediacam, 369 were tested for CMV. There were no significant differences in the following characteristics between infants tested and not tested for CMV in the Yaounde sites: feeding practice, recruitment site, maternal marital status, and infant age at inclusion. However, the proportion of uninfected infants born to HIV-uninfected mothers was higher in “the not screened” than “screened” for CMV group (47.1% (48/102) vs 29.0% (107/369); *p* < 0.001). Table 1 reports and compares the characteristics of the 369 infants according to HIV status and mode of inclusion: 41 HIV-infected infants followed from birth (group 1i), 105 HIV-infected infants diagnosed later in life before age of 7 months (group 3i), 116 HIV-uninfected infants born to HIV-infected mothers (group 1ni), and 107 HIV-uninfected infants born to HIV-uninfected mothers (group 2ni). These groups were significantly different when maternal education level and breastfeeding were considered. The proportion of breastfed infant was 99.1% among HIV^-^ infants born to HIV uninfected mothers, 58.7% among HIV^+^ infants not followed from birth but diagnosed before age of 7 months, 27.5% among HIV^+^ infants followed from birth, and 12.9% among HIV^-^ infants born to HIV^+^ mothers.

**Table 1:** Comparison of characteristics of infants screened for CMV infection at inclusion into phase 2 of the ANRS-PEDIACAM study according to HIV status and mode of inclusion, 2007–2013, Cameroon

	HIV-infected infants						HIV-uninfected infants							
	Followed from birth (group 1i, *n* = 41)		Diagnosed before 7 months of age (group 3i, *n* = 105)		Total (*N* = 146)		Born to HIV-infected mothers (group 1ni, *n* = 116)		Born to HIV-uninfected mothers (group 2ni, *n* = 107)		Total (*N* = 223)		*p*-values	
	n	%	n	%	n	%	n	%	n	%	n	%	P1	P2
Recruitment site														
MCC-CBF	25	61.0	70	66.7	95	65.1	74	63.8	64	59.8	138	61.9	0.76	0.61
EHC	16	39.0	35	33.3	51	34.9	42	36.2	43	40.2	85	38.1		
Gender: Female	26	63.4	55	52.4	81	55.5	61	52.6	54	50.5	115	51.6	0.56	0.53
Maternal marital status			*(n = 104)*		*(n = 145)*									
Married/Cohabiting	28	68.3	63	60.6	91	62.8	76	65.5	71	66.4	147	65.9	0.86	0.67
Single/Divorced/Widow	13	26.8	41	36.5	54	33.8	40	31.9	36	31.8	76	31.8		
Maternal Level of education	*(n = 40)*		*(n = 104)*		*(n = 144)*		*(n = 114)*				*(n = 221)*			
Higher education	5	12.5	3	2.9	8	5.6	28	24.6	49	45.8	77	34.8	<.001	<.001
Secondary education	27	67.5	63	60.6	90	62.5	61	53.5	53	49.5	114	51.6		
None or primary education	8	20.0	38	36.5	46	31.9	25	21.9	5	4.7	30	13.6		
Maternal professional activity	*(n = 39)*		*(n = 104)*		*(n = 143)*		*(n = 113)*				*(n = 220)*			
Paid activity	18	46.2	32	30.8	50	35.0	58	51.3	47	43.9	105	47.7	0.02	0.02
Housewife/student/unemployed	21	53.8	72	69.2	93	65.0	55	48.7	60	56.1	115	52.3		
Existence of a functional fridge at home	*(n = 39)*		*(n = 99)*		*(n = 138)*		*(n = 111)*		*(n = 103)*		*(n = 214)*			
Yes	19	50.0	34	34.3	53	38.7	57	51.4	69	67.0	126	58.9	<.001	<.001
No	19	50.0	65	65.7	84	61.3	54	48.6	34	33.0	88	41.1		
Premature delivery			*(n = 85)*		*(n = 126)*									
≥ 37 weeks	37	90.2	72	84.7	109	86.5	110	94.8	94	87.9	204	91.5	0.10	0.20
< 37 weeks	4	9.8	13	15.3	17	13.5	6	5.2	13	12.1	19	8.5		
Mode of delivery			*(n = 102)*		*(n = 143)*									
Vaginal	40	97.6	100	98.0	140	97.9	102	87.9	95	88.8	197	88.3	0.01	<.001
Cesarean	1	2.4	2	2.0	3	2.1	14	12.1	12	11.2	26	11.7		
Feeding practice	*(n = 40)*		*(n = 104)*		*(n = 144)*									
No breastfeeding	29	72.5	43	41.3	72	50.0	101	87.1	1	0.9	102	45.7	<.001	0.49
Any history of breastfeeding	11	27.5	61	58.7	72	50.0	15	12.9	106	99.1	121	54.3		
CMV infection														
Negative	27	65.9	33	31.4	60	41.1	98	84.5	58	54.2	156	70.0	<.001	<.001
Positive	14	34.1	72	68.6	86	58.9	18	15.5	49	45.8	67	30.0		

*MCC-CBF* Mother and Child Center of the Chantal Biya Foundation; *EHC* Essos Hospital Center in Yaounde P1: *p*-value for comparison of characteristics between groups (1i, 3i, 1ni, 2ni). P2: *p*-value for comparison of characteristics between HIV-infected and HIV-uninfected infants

The overall median age at CMV screening was 4.0 months [Interquartile range (IQR): 3.4–4.9], and was 2.6 months [IQR: 2.2–3.4] for HIV^+^ infants followed from birth to 4.3 months [IQR: 3.2–5.8] for HIV^+^ infants not followed from birth (Fig. 1).

### Prevalence and factors associated with CMV infection at baseline

The overall CMV prevalence was 41.5% (95% CI: 36.4–46.7): 15.5% (9.5–23.4) for HIV^-^ infants born to HIV^+^ mothers, 34.1% (20.1–50.6) for HIV^+^ infants followed from birth, 45.8% (36.1–55.7) for HIV^-^ infants born to HIV^-^ mothers and 68.6% (58.8–77.3) for HIV^+^ infants not followed from birth but diagnosed before age of 7 months (*p* < 0.01) (Table 1). The prevalence of CMV infection and the median CMV viral load were both significantly higher among HIV^+^ (58.9%; 3.7 log copies/mL [IQR: 2.5–3.9]) than HIV^-^ (30.0%; 2.8 log copies [IQR; 2.1–3.4]) infants.

In the HIV^+^ groups of infants, breastfeeding was more prevalent among infants who were CMV^+^ than CMV^-^ (70.6% vs 20.3%; *p* < 0.001); CMV^+^ infants compared to CMV^-^ infants were more underweight (50.0% vs 28.3% with weight-for-age Z scores < -2SD; *p* = 0.01); more sick (55.8% vs 26.7% with HIV clinical stage 3 or 4; *p* < 0.001); and more immunosuppressed (74.4% vs 47.5% with lymphocytes TCD4 < 25%; *p* = 0.002) respectively.

Significant differences were observed when comparing CMV-infected infants, who were exposed but uninfected with HIV versus those who were unexposed and uninfected with HIV in the following characteristics: female gender (83.3% vs 49%, *p* = 0.02), proportion of mothers who attended higher education (22.2% vs 36.7%, *p* < 0.01) and proportion of breastfed infants (38.9% vs 100%, *p* < 0.01). Apart from gender, the same differences were observed between the two groups among the CMV-uninfected group (Table 2).

**Table 2:** Comparison of characteristics between HIV-exposed uninfected and HIV-unexposed-uninfected infants according to CMV status at inclusion into phase 2 of the ANRS 12140-Pediacam study, 2007–2013, Cameroon

	CMV-infected infants					CMV-uninfected infants				
	HIV-exposed uninfected		HIV-unexposed-uninfected			HIV-exposed uninfected		HIV-unexposed-uninfected		
	N	% (n)	N	% (n)	*p*-value*	N	% (n)	N	% (n)	*p*-value*
Study site: Essos Hospital Center	18	33.3 (6)	49	42.9 (21)	0.67	98	36.7 (36)	58	37.9 (22)	1.00
Gender: Female	18	83.3 (15)	49	49.0 (24)	0.02	98	46.9 (46)	58	51.7 (30)	0.68
Marital status: Alone (single or divorced or widow)	18	22.2 (4)	49	34.7 (17)	0.50	98	36.7 (36)	58	32.8 (19)	0.74
Electricity supply at home: Yes	18	100.0 (18)	48	97.9 (47)	1.00	94	98.9 (93)	57	100 (57)	1.00
Maternal level of education: Higher education	18	22.2 (4)	49	36.7 (18)	<0.01	96	25.0 (24)	58	53.4 (31)	<0.01
Maternal age at inclusion: > = 25 years	18	77.8 (14)	49	69.4 (34)	0.71	97	79.4 (77)	58	81 (47)	0.97
Professional activity of the mother: Paid	17	58.8 (10)	49	42.9 (21)	0.39	96	50.0 (48)	58	44.8 (26)	0.65
Mode of delivery: Cesarean section	18	11.1 (2)	49	10.2 (5)	1.00	98	12.2 (12)	58	12.1 (7)	1.00
Breastfeeding: Yes	18	38.9 (7)	49	100 (49)	<0.01	98	8.2 (8)	58	98.3 (57)	<0.01
Prematurity: <37 weeks	18	16.7 (3)	49	10.2 (5)	0.67	98	3.1 (3)	58	13.8 (8)	0.02
Hemoglobin level: < 9 g/dl	18	5.6 (1)	49	4.1 (2)	1.00	98	3.1 (3)	58	6.9 (4)	0.43
Age at CMV screening: > = 4 months	18	38.9 (7)	49	46.9 (23)	0.76	98	64.3 (63)	58	50.0 (29)	0.11
T CD4 lymphocytes : < 25%	18	11.1 (2)	49	12.2 (6)	1.00	96	3.1 (3)	58	10.3 (6)	0.08
Z-score weight for age: ≤2 SD	18	0.0 (0)	49	2.0 (1)	1.00	98	1.0 (1)	58	0.0 (0)	1.00
Hospitalization at or before inclusion: Yes	18	0.0 (0)	49	4.1 (2)	1.00	98	3.1 (3)	58	1.7 (1)	1.00

* *p*-value based on Fisher’s exact test

Table 3 shows in bivariable analysis that among HIV^+^ infants, CMV infection was associated with: infant inclusion group (frequent among HIV^+^ infants included later but before 7 months); recruitment site (being less frequent in the Essos Hospital Center than at the MCC-CBF); married or cohabiting maternal status; and history of breastfeeding. Infants screened for CMV after 4 months more likely to be CMV infected than those screened at a younger age. All these associations, except age at CMV screening, remained significant and of the same magnitude in multivariable logistic regression analysis. Inclusion group and breastfeeding act as confounders of the relation between age at CMV screening and CMV infection. No interaction was identified between these factors or with other factors included in the multivariable analysis. None of the surrogates of economic status (water supply at home, presence of a functional fridge at home, electricity at home, professional activity of the mother and maternal level of education) listed in Table 3 was associated with CMV infection in either bi or multivariable analysis.

**Table 3:** Factors associated with CMV infection in HIV-infected infants enrolled into phase 2 of the ANRS-PEDIACAM study, Cameroon, 2007–2013 (bivariable and multivariable logistic regression analysis)

	CMV infected infants							
	Bivariable analysis *N* = 146					Multivariable analysis *N* = 142		
		% (n) 58.6 (86)	cOR (95% CI)		*p*	aOR (95% CI)		*p*
HIV infected group in Pediacam (*n* = 146)								
Identified during first week of life	41	34.1 (14)	1			1		
Identified later at diagnosis before 7 months of age	105	68.6 (72)	4.2	(1.8–9.8)	<.01	2.9	(1.0–7.8)	0.04
Recruitment site (*n* = 146)								
MCC-CBF	95	66.3 (63)	1			1		
EHC	51	45.1 (23)	0.4	(0.2–0.9)	0.02	0.4	(0.2–0.9)	0.03
Gender (*n* = 146)								
Female	81	55.6 (45)	1					
Male	65	63.1 (41)	1.4	(0.7–2.8)	0.45			
Mode of delivery (*n* = 143)								
Vaginal	140	59.3 (83)	1					
Cesarean	3	0.0 (0)	0.0	(0.0–1.7)	0.07			
Age at CMV screening (*n* = 146)								
< 4 months	82	48.8 (40)	1			1		
> =4 months	64	71.9 (46)	2.7	(1.3–5.7)	<.01	1.3	(0.5–3.2)	0.62
Feeding practice (*n* = 144)								
No breastfeeding	72	34.7 (25)	1			1		
Any history of breastfeeding	72	83.3 (60)	9.2	(4.0–22.6)	<.01	6.7	(2.8–15.9)	<.01
Water supply at home (*n* = 139)								
Yes	61	60.7 (37)	1					
No	78	56.4 (44)	0.8	(0.4–1.8)	0.74			
Existence of a functional fridge at home(*n* = 137)								
Yes	53	60.4 (32)	1					
No	84	58.3 (49)	0.9	(0.4–2.0)	0.95			
Electricity supply at home (*n* = 139)								
Yes	117	56.4 (66)	1					
No	22	68.2 (15)	1.7	(0.6–5.2)	0.43			
Professional activity of the mother (*n* = 143)								
Housewife/student/unemployed	93	63.4 (59)	1			1		
Paid activity	50	52.0 (26)	0.6	(0.3–1.3)	0.25	0.7	(0.3–1.6)	0.33
Maternal level of education (*n* = 144)								
Higher education	8	37.5 (3)	1			1		
Secondary education	90	55.6 (50)	2.1	(0.4–14.1)	0.13	1.2	(0.2–6.6)	0.96
None or primary education	46	69.6 (32)	3.7	(0.6–27.2)		1.3	(0.2–8.4)	
Maternal marital status (*n* = 145)								
Single/divorced/widow	54	42.6 (23)	1			1		
Married/cohabiting	91	68.1 (62)	2.9	(1.4–6.1)	<.01	3.4	(1.4–8.2)	<.01
Maternal age at inclusion (*n* = 144)								
> = 25 years	102	55.9 (57)	1					
< 25 years	42	64.3 (27)	1.4	(0.6–3.2)	0.46			
HIV-infected mothers’ ART status (*n* = 141)								
HAART started before or during pregnancy	14	57.1 (8)	1					
ART prophylaxis	48	50.0 (24)	0.8	(0.2–2.9)	0.27			
Not treated	79	64.6 (51)	1.4	(0.4–5.0)				

The variable “Mode of delivery” was not included in the multivariable analysis because of the small number of Cesareans

*MCC-CBF* Mother and Child Center of the Chantal Biya Foundation; *EHC* Essos Hospital Center; *cOR* Crude Odds ratio; *aOR* adjusted Odds Ratio; *CI* Confidence Interval

### Relation between CMV infection and various disease outcomes after 1 year of early cART in HIV-infected infants

Among the 132 HIV-infected infants who initiated cART, 58.3% (*n* = 77) had CMV co-infection. Other than continuation of the cART, no specific anti-CMV treatment was given and no major clinical event was recorded. Overall, 99 HIV infected infants (61 CMV^+^ and 38 CMV^-^) were retested for CMV after a median duration of cART of 7.1 months (range [4.8–9.5], similar in both groups). The mean number of CMV tests was 2 (range [1–4]). CMV was undetectable on retest in 37 (60.7%) of the 61 initially HIV^+^CMV^+^ infants. CMV was detected on retest in 8 (21.1%) of the 38 initially HIV^+^CMV^-^ infants.

The proportion of infants not followed from birth was higher in CMV^+^ than CMV^-^ group (81.8% vs 56.4%; *p* = 0.001). cART was initiated later in CMV^+^ infants than CMV^-^ infants (median age of 4.7 months, IQR: 3.5–6.2 vs 3.6 months, IQR: 2.8–4.8; *p* = 0.03).

Kaplan-Meier estimates of probability of death after 1 year of cART treatment did not differ significantly between CMV^+^ HIV^+^ and CMV^-^ HIV^+^ infants [18.5%, 95% CI: 11.4–29.2 vs 20.3, 95% CI: 11.8–33.7]; log rank test, *p* = 0.75). Taking into consideration age at cART initiation (<4 months (early) and ≥4 months (late)), the survival rate were similar for HIV/CMV co-infected treated early [0.129, 95% CI: 0.050–0.308], HIV/CMV co-infected treated later [0.225, 95% CI: 0.128–0.378], HIV infected/CMV uninfected treated early [0.249, 95% CI: 0.138–0.424], and HIV infected/CMV uninfected treated later [0.111, 95% CI: 0.030–0.376] (log rank test, *p* = 0.45) groups. This was also true of death/loss to follow up as composite outcomes (*p* = 0.38). The proportion of cases with adequate HIV RNA viral suppression was similar in HIV-CMV co-infected and HIV infected groups (75.5% vs 61.5%; *p* = 0.17). The mean difference in CD4 percentage (change between initiation of cART and the last follow up) was similar CMV+ and CMV- groups (10.97%, standard deviation (SD): 13.66% vs 6.88%; SD: 15.25%; *p* = 0.15).

## Discussion

Little is known about the prevalence of early CMV infections according to the HIV status of infants in resource-limited settings and even less about its potential role in HIV progression in infants treated early with cART. We compared the prevalence of CMV infection in HIV-infected and -uninfected infants born to HIV-infected mothers and a control group of infants born to HIV-negative mothers, all in an urban area of Cameroon. We also report the first comparison of the prognosis of HIV infection during first year after early cART initiation between HIV^+^ infants with and without CMV co-infection.

Consistent with other studies, we observed that CMV infection prevalence was higher among HIV-infected than non-infected infants [6, 9, 14]. One study showed a higher prevalence of CMV at birth in Kenyan HIV-infected than HIV-exposed uninfected infants but did not find any difference by 3 months of age [9]. In another study, CMV prevalence was similar at birth but significantly different by 6 months [6]. CMV viremia peaks at 3–4 months of age [15], indicating that direct CMV diagnostic testing could be particularly sensitive at this age. Note that most infants in sub-Saharan Africa are already infected with CMV by 1 year of age [1].

We report here that CMV was associated with breastfeeding. This was the only single factor independently associated with CMV infection among HIV-uninfected infants (born to HIV-infected or HIV-uninfected mothers, data not shown). In Cameroon, 98% of children under 6 months old are breastfed, either getting breast milk exclusively or in mixed feeding [16]. CMV can be shed into breast milk and infects breastfeeding children [17]. CMV shedding into breast milk has been detected as early as the first week after delivery and peaks between 3 to 5 weeks after delivery [18].

Most breastfed infants of HIV-infected mothers in resource-limited settings are infected with CMV by 24 weeks of age [19]. The two groups in our study with high proportions of breastfed infants (HIV^-^ infants born to uninfected mothers and HIV^+^ infants not followed from birth but diagnosed at an age <7 months) also had high prevalence of CMV infection (45.8 and 68.6% CMV infection, respectively). This suggests a postnatal transmission of CMV through breast milk. Surprisingly, a recent study did not find an association between breastfeeding and CMV infection in infants [11], but this may have been due, in part, to the limited number of CMV-infected infants they included.

Non-single marital status of the mother was also associated with a higher risk of CMV infection. Frequent sexual exposure, as part of the relationship of a couple may increase the risk of maternal infection and re-infection with CMV and subsequently its transmission to the newborn. Very different observations have also been reported [20, 21], where CMV infection was more frequent among children born to young and unmarried women.

We found no evidence that CMV infection was associated with the lack of PMTCT prophylaxis in mothers of HIV-infected infants. Similarly, Manicklal and colleagues did not find any relationship between ARV prophylaxis and congenital CMV infection in HIV-infected infants [11]. However other studies have suggested that there is an effect of ARV prophylaxis on CMV infection [7, 22, 23], cART reduces serological biomarkers of inflammation and immune activation [24, 25]. ARV could impede CMV reactivation in HIV-infected pregnant women and thereby reduce the possibility of CMV transmission to infants born to mothers who took prophylactic regimens. The difference observed in our study could be explained by the fact that we carried out our CMV analyses starting at a median age of 4 months; consequently we could not distinctively identify congenital infections and therefore could not establish the link between prophylactic cART during pregnancy and congenital CMV infections. Bivariable analysis showed that among HIV^+^ infants, CMV infection was associated with: infant inclusion group; recruitment site, married or cohabiting maternal status, history of breastfeeding and age at CMV screening. All these associations, except age at CMV screening, remained significant and of the same magnitude in multivariable logistic regression analysis. Though the identification and inclusion of infants of group 3i (*n* = 105) were in average done later than all other groups, they were in details not all diagnosed at 7 months as 47 were diagnosed at less 4 months and 58 at 4 months or later. In contrast, infants of group 1i were mostly diagnosed at less than 4 months. Only 6 out of 41 were diagnosed at 4 months or later. Bringing these two groups of HIV infected infants together probably had an impact on the effect age at CMV screening could have with CMV infection in multivariable analyses.

Among HIV-infected infants, those with CMV co-infections were mostly immunosuppressed, showed poorer growth, and had more advanced disease. A rapid progression of HIV disease in the presence of CMV co-infection has been described [5, 6, 26–28]. Kovacs et al. observed that infants with both HIV and CMV infections were more likely than those with HIV infection alone to have class C symptoms (severe immune suppression), to have died by or to have HIV-1 associated encephalopathy by 18 months [6]. Therefore, we considered in our analyses that clinical signs associated with CMV infection in infants were consequences rather than causes of the infection and thus these signs were not included in the multivariable analysis to identify independent risk factors associated with CMV.

We compared the two groups of HIV-infected infants (CMV infected and CMV uninfected) 1 year after initiation of cART. This subgroup of HIV infected infants (CMV+ and CMV-) under cART was particularly studied in line with the objective of Pediacam which was set up to study all factors that could be related with early treatment of HIV in resource limited setting like Cameroon. Normally, CMV infection is not a major problem in HIV-uninfected children, as most of them remain asymptomatic. However, CMV has been described to have a negative impact in disease progression in HIV-infected infants. In resource limited settings CMV treatment is not always available and even when available could be very toxic. Here, we sought to know if early cART could reduce HIV disease progression and/or improve survival in these children. These two groups did not differ significantly regarding the probability of death, composite outcomes (death and lost to follow-up), adequate viral suppression and mean change in CD4 percentage since cART initiation. This is consistent with early cART having counterbalanced the potential negative role of CMV/HIV co-infections through immune restoration and control of HIV replication [29]. O’Sullivan observed that adult patients (mean age, 38 years) who were HIV/CMV co-infected and treated with cART with no specific anti-CMV therapy showed a significant drop in CMV viral load [30]: after 1 year, cART was associated with the complete suppression of CMV viremia in all patients who returned for follow-up (23 of 38). We observed no such effect in HIV/CMV co-infected infants on cART. However, 21.1% (8/38) of early treated HIV-infected infants who were CMV negative at inclusion in Pediacam (before cART) presented subsequent positive tests. This may correspond to recent infection, common in HIV-uninfected infants.

The strength of this study is the inclusion of four groups of infants based on their HIV infection and/or exposure status; all were recruited at the same sites the same time frame, and were followed in a similar manner. However, there are also some limitations. The prevalence estimates cannot be generalized to other regions or rural areas in Cameroon, as all the mothers were living in urban areas of central Cameroon. Blood samples were not collected immediately after delivery and for this reason it was not possible to identify *in-utero* transmissions of CMV. Also, 20% of the infants were not tested for CMV on inclusion into phase 2, and their characteristics did not differ from those tested. According to these observations, we hypothesized that this group of CMV screened infants participating in this study could be representative of the study population. However there was a higher proportion of non-tested infants among HIV-uninfected infants born to HIV-uninfected mothers. Consequently, any selection bias should be limited to results concerning the HIV-infected group.

## Conclusion

We observed a high prevalence of CMV infection among HIV-infected infants with associated breastfeeding exacerbating this prevalence. However early initiation of cART was shown to limit the negative impact of CMV on HIV disease progression in CMV/HIV co- infected infants even in the absence of anti-CMV specific treatment. This study underscores the benefits of early cART in the case of CMV/HIV coinfection and further reinforces the recommendations to offer early antiretroviral therapy to all HIV infected infants.

## Abbreviations

cART: Combined antiretroviral therapy
CMV: Cytomegalovirus
DNA: Deoxyribonucleic acid
HAART: Highly Active Antiretroviral Therapy
HCE: The Hospital Center Essos
HIV: Human Immunodeficiency Virus
IQR: Interquartile range
LH: Laquintinie Hospital in Douala
MCC-CBF: The Mother and Child Center of the Chantal Biya Foundation
PCR: Polymerase Chain Reaction
PMTCT: Prevention of mother-to-child transmission
RNA: Ribonucleic acid
